# Outcomes of Tympanoplasty with an Autologous Two-Piece Perichondrium-Cartilage Graft in a Tertiary Care Setting

**DOI:** 10.3390/jcm14082600

**Published:** 2025-04-10

**Authors:** Marie Reynders, Dylen Philips, Kelsey Van Den Houte, Lynn Van Der Sypt, Camille Levie, Ina Foulon

**Affiliations:** 1 Department of Otorhinolaryngology and Head & Neck Surgery, University Hospital of Brussels, 2610 Brussels, Belgium; dylen.philips@zas.be (D.P.); kelsey.vandenhoute@uzbrussel.be (K.V.D.H.); lynn.vandersypt@uzbrussel.be (L.V.D.S.); camille.levie@uzbrussel.be (C.L.); ina.foulon@uzbrussel.be (I.F.); 2 MIPI, Vitality Research Group, Vrije Universiteit Brussel, Laarbeeklaan 103, 1090 Brussel, Belgium; 3 Ear-Nose-Throat Department, European Institute of Otorhinolaryngology, Head and Neck Surgery, 2610 Antwerp, Belgium

**Keywords:** tympanoplasty, cartilage, fascia, hearing loss, perforation

## Abstract

**Background/Objectives**: This study evaluates the anatomical and functional outcomes of type 1 tympanoplasty using an autologous two-piece perichondrium-cartilage (CP) graft in pediatric and adult patients with tympanic membrane (TM) perforations. **Methods**: A retrospective review of 74 patients (59 children, 15 adults) undergoing type 1 tympanoplasty with CP by a single surgeon (IF) was conducted. Preoperative and postoperative audiological outcomes, perforation size, prognostic factors, and complications were analyzed. Success was defined as an intact TM and an air–bone gap (ABG) < 20 dBHL at 12 months postoperatively. **Results**: TM closure was achieved in 93.2% of patients, with 93.1% attaining an ABG < 20 dBHL. The combined success rate was 86.3%, with no significant differences between children and adults. Larger perforations (>50%) had significantly lower closure rates (55.6% vs. >97%, *p* < 0.002). Children who underwent prior adenoidectomy had significantly higher success rates (*p* = 0.04). **Conclusions**: Tympanoplasty with a CP graft provides high success rates in both children and adults. The procedure can be performed from age five, considering patient cooperation. In children, simultaneous adenoidectomy is recommended if significant adenoid hypertrophy is present to optimize outcomes. Larger perforations were associated with reduced success, while age had no significant impact.

## 1. Introduction

The repair of tympanic membrane (TM) perforations is a common procedure in otology. The prevalence of TM perforations is significant, affecting approximately 2.1% of the American population [1]. TM perforations can impact hearing, swimming ability, and can cause infections ranging from mild to severe, with potential chronic or even life-threatening complications. The aim of this surgery is, first and foremost, to achieve a safe ear and secondly, to restore hearing loss.

Debates among otologists persist regarding the optimal technique and reconstruction graft material. The most common graft materials described in literature are fascia and cartilage, with or without perichondrium. The consistency of fascia is similar to that of the TM but is known to undergo postoperative shrinkage or retraction, which may lead to reperforation [2,3]. As a result, many centers consider cartilage-perichondrium (CP) to be the most successful reconstruction material [4,5,6]. The main reason for this is that CP is thicker and more resistant to negative middle ear pressure and Eustachian tube dysfunction. On the other hand, CP grafts do present with some challenges, namely difficult postoperative middle ear evaluation [4,7]. The thickness and rigidity of the CP graft, compared to a normal eardrum, have raised concerns about potential auditory impairment. However, several studies contradicted this perception [4,8,9,10,11].

Tympano-ossicular allografts are an alternative reconstruction material, which restore both the eardrum and the malleus to their original status. In the case of mobile stapes, this approach tends to give better hearing outcomes compared to cartilage-perichondrium (CP) [12]. However, this technique is not widely available, as it requires a prelevation team and tissue bank. Consequently, this material may not be the preferred choice for simple TM perforations without ossicular chain damage.

In our prior study [13] on type 1 tympanoplasty with fascia grafts in children, we reported a success rate of 80.2%, defined as an intact TM after 12 months with an air–bone gap (ABG) of <20 decibel hearing loss (dBHL). TM closure was achieved in 86.5% of cases. Larger perforations, particularly those exceeding 50% of the TM surface area, were associated with poorer outcomes, with success rates as low as 42.9% [13]. While these results are favorable, superior outcomes have been documented in the literature when using CP [14,15]. These findings led to the exploration and implementation of a type 1 tympanoplasty with CP, aiming to improve surgical outcomes. While several studies have compared fascia and cartilage grafts in tympanoplasty, many have been conducted by different surgeons, introducing a potential bias due to varying surgical expertise [16,17,18,19]. Additionally, some studies have included patients with more complex conditions, such as those requiring mastoidectomy or only presenting with large perforations, making direct comparisons challenging [1,20].

This study aims to evaluate the anatomical and functional outcomes of type 1 tympanoplasty using an autologous two-piece CP graft in a cohort of pediatric and adult patients with TM perforations, free of other middle ear pathologies (e.g., cholesteatoma, chain disruptions, chronic otitis media). Additionally, it examines potential confounding factors that may influence the success of the procedure. Lastly, to provide a more objective comparison of success rates between fascia and cartilage graft tympanoplasty, all surgeries were performed by the same experienced surgeon.

## 2. Materials and Methods

### 2.1. Study Design

This was a retrospective study examining all records from a pediatric and adult population who underwent type 1 tympanoplasty with CP performed by a single surgeon (IF) at UZ Brussel. Patients were included from July 2014 to November 2023. This study was approved by the medical ethics committee of UZ Brussel prior to its commencement and was assigned the Belgian Unique Number: 1432024000042.

### 2.2. Subjects

Patients were included if they underwent type 1 tympanoplasty with CP for a persistent TM perforation and had a minimum follow-up period of 12 months at the time of analysis. All patients included in this study underwent the same surgical procedure. Patients with an associated cholesteatoma, ossicular chain defect, chronic otitis media, or TM retraction without perforation were excluded. The population was divided into four age groups: <8 years, 8–12 years, 13–17 years, and ≥18 years. This division was based on the anatomical maturation of the Eustachian tube and existing literature [13].

### 2.3. Prognostic Factors and Complications

The prognostic factors analyzed included pre-existing allergies, if the surgery was a revision, condition of the contralateral ear, prior ventilation tube placement, risk factors (e.g., cleft palate, mental retardation, and immunological disorders), post- and perioperative ear discharge and the surgical side. The size and location of the TM perforation were estimated intraoperatively by the surgeon using otomicroscopy. Any complications and/or discomfort during or after surgery were recorded.

### 2.4. Audiological Evaluation

Audiological assessments were conducted preoperatively and postoperatively (at 6 weeks and 12 months) for frequencies ranging from 125 to 8000 Hz for air conduction and 250 to 4000 Hz for bone conduction. The details of this assessment protocol were previously described [13].

### 2.5. Surgical Technique

**Figure 1:**
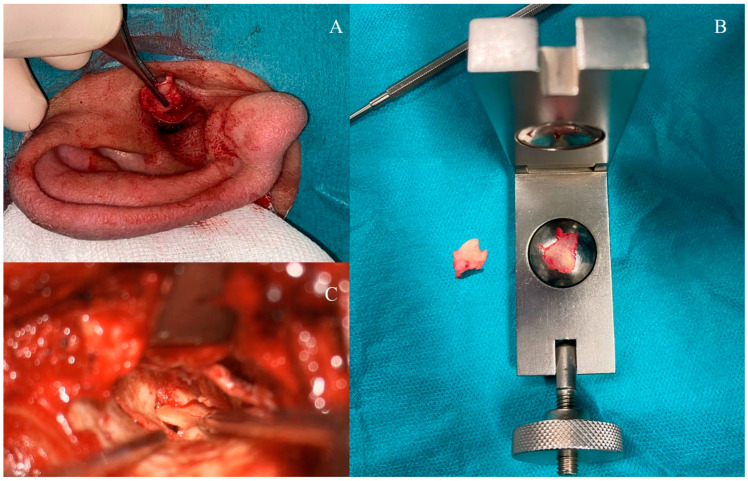
(**A**) Harvesting tragal cartilage-perichondrium graft. (**B**) Preparing the cartilage-perichondrium graft. (**C**) Transmeatal repairing of the tympanic membrane defect.

All patients underwent surgery following a standardized protocol as previously described [13], performed by the same surgeon (IF). The sole variation in technique was the choice of graft material: in every case, CP was utilized to close the TM perforation. In this series, all procedures were carried out under general anesthesia using a retro-auricular approach. The graft material was consistently harvested from the tragus. Prior to elevating the TM and inspecting the middle ear, the edges of the perforation were carefully refreshed and revitalized. Sliced cartilage was used to close the perforation partially or completely. In all cases, perichondrium was layered over the cartilage and beneath the TM to ensure coverage of the cartilage and any residual gaps (Figure 1). The CP was supported with gelfoam to prevent medialization. The neotympanum was covered with a silastic sheet. A single IV dose of intraoperative antibiotics was administered to every patient. The external auditory canal was packed with gelfoam soaked in a suspension of oxytetracycline and hydrocortisone. After seven days, the ear packing and silastic sheet were removed, and topical neomycin drops were prescribed for an additional five days to promote healing. Ear protection against water was advised and physical exercise was discouraged for at least one month postoperatively.

### 2.6. Definition of Success

The surgery was considered successful if the TM remained intact at the last postoperative visit, verified by micro-otoscopy (Zeiss, Jena, Germany) and tympanometry during outpatient follow-up, and if the ABG was less than 20 dB HL on pure tone audiometry (PTA).

### 2.7. Statistical Analysis

The statistic software R version 4.2.1 was used to perform the statistical analyses. Summary statistics of patient characteristics were presented as frequency (*n*), percentages (%), means with standard deviations and medians with range. To test the relationship between possible prognostic factors on tympanoplasty, a chi-square test or Fisher’s exact test was used. Pre-planned sub analysis was made in the children group (<18 years) and adult group (≥18 years). The two-proportion z-test of Fisher’s exact test was used to see if the difference between the proportions of adults and children were statistically significant. All tests were performed at a significant level of 0.05. Data normality was assessed using the Kolmogorov–Smirnov test of normality.

## 3. Results

### 3.1. Subjects

Data from 113 patients that underwent type 1 tympanoplasty with CP was analyzed. Twenty-three patients were excluded due to the co-performance during surgery of attico(antro)mastoidectomy, an associated cholesteatoma or chain reconstruction. Fourteen patients were excluded because the indication for surgery was a retraction pocket rather than a perforation. Two patients were excluded because the procedure was endoscopically performed. The remaining 74 patients fulfilled all the inclusion criteria and were included in this study. In one child, there was no data available on the ABG postoperative, this patient was therefore not included in the analysis regarding the postoperative ABG. The mean age of the total group at surgery was 14.8 (range 5–69 years). The subgroup of children consisted of 59 patients (79.7%), with a mean age of 8.2 (range 5–16 years). The children were divided into three age groups: <8 years (31 patients), 8–12 years (21 patients) and 13–17 years (7 patients). Fifteen adults (20.3%) were included with a mean age of 40.7 (range 18–69) years. The mean follow-up after surgery of the total group was 23.8 (range 12–103) months.

### 3.2. Results of Type 1 Tympanoplasty with CP

In the overall cohort, tympanoplasty with CP achieved a closed TM in 69 patients (93.2%) and an ABG of <20 dBHL in 68 patients (93.1%) at the 12-month follow-up. A successful outcome, defined as both a closed TM and an ABG < 20 dB HL, was recorded in 63 patients (86.3%). On average, the ABG improved by 11.4 dB HL, and the PTA improved by 11.2 dB HL. In two patients the ABG did not change (2.7%) and in eight patients the hearing worsened (10%), with a median decline of 5.7 dB HL (range: 1–14 dB HL). The hearing outcomes of the cohort of this study were compared to our previous cohort operated with fascia, and no statistical difference was found (ABG fascia: 11.3 dB HL, *p* = 1) [13]. Statistical analysis revealed no significant differences between children and adults in terms of achieving a closed TM, ABG < 20 dBHL, or overall success rates (*p* > 0.05). Detailed outcomes by subgroup (children vs. adults) are summarized in Table 1.

### 3.3. Influence of Age

As illustrated in Table 1, children and adults did not show significant differences on tympanoplasty outcomes. When examining the pediatric subgroups, the rates of closed TM were 90.3% (28/31) for children under 8 years, 95.2% (20/21) for those aged 8–12 years, and 100% (7/7) for those aged 13–17 years (*p* = 0.59). Similarly, the 12-month success rate (closed TM and ABG < 20 dBHL postoperative) showed no statistically significant variation across pediatric age groups, with rates of 83.3% (25/30), 90.5% (19/21), and 85.7% (6/7) for children under 8, 8–12, and over 12 years, respectively (*p* = 0.78). These findings indicate that age, whether considered within pediatric subgroups (Table 2) or compared between children and adults (Table 1), does not significantly affect the surgical success of tympanoplasty.

### 3.4. Prognostic Factors for Successful Tympanoplasty

The examined prognostic factors for a successful tympanoplasty are listed in Table 3. Whether the patients had pre-existing allergies, if the surgery was a revision, condition of the contralateral ear, prior ventilation tube placement, risk factors (e.g., cleft palate, mental retardation, immunological disorders), post- and perioperative ear discharge or the surgical side, showed no significant effect on outcomes (Table 3). All patients with symptomatic allergies were treated with intranasal cortisone. However, no data were collected regarding the use of antihistamines or patient compliance with allergy treatment. These findings remained consistent when analyzed separately in children and adults. In the pediatric subgroup, the statistical difference in successful operation was significant between the children that did not undergo adenoidectomy (84%) compared to children that did undergo prior adenoidectomy (100%, *p* = 0.04). In the adult subgroup, the effect of smoking was analyzed, but no difference in success rates was observed between smokers and non-smokers (*p* = 0.15).

### 3.5. Size and Location of the Perforation

In the total cohort, the findings show that when the perforation was larger than 50%, the chance of a closed TM was lower (55.6%) compared to smaller perforations. In cases with perforations < 25% and 25–50%, the chances of successful closure were 100% and 97.4%, respectively. A statistically significant effect of perforation size on the overall success rate was observed (*p* < 0.002). When analyzed separately in children, similar results were found (Table 4). However, in adults, no statistical difference was observed, likely due to the small sample size. If the perforation was in the anterosuperior quadrant, the success rate was lower (12/15, 80%) compared to other locations (56/58, 96%), though the difference was not significantly different (*p* = 0.06).

### 3.6. Complications Other than Perforations

In this study, complications following tympanoplasty were observed in 45% of the total cohort, 38.9% of children, and 60% of adults. The majority of the complications were observed >1 month postoperatively. The most common complications in both groups were single episodes of ear discharge (20.3%) and granulation tissue formation (10.8%). These complications were effectively treated with localized antibiotics and corticosteroid drops. Patients with complications showed a trend toward lower success rates compared to those without (90.3% vs. 100%, *p* = 0.07), though the difference was not statistically significant. At the final follow-up visit, complications persisted in only four patients (5%), including otitis media with effusion (OME), granulation tissue, keloid formation, and numbness around the ear. In the children subgroup, success rates were 91.3% in those with complications and 100% for those without, also not statistically significant (*p* = 0.53). In adults, the presence of complications did not significantly affect outcomes (88% vs. 100%, *p* = 1). The distribution and types of complications across all patient groups are summarized in Table 5.

## 4. Discussion

In this study, TM closure was achieved in 93.2% of children after tympanoplasty with CP. The success rate, defined as a closed TM with an ABG < 20 dBHL, was 86.2%. Our previous study on tympanoplasty with fascia in children, performed by the same surgeon, reported lower rates, with 86.5% achieving closure and 80.3% meeting the success criteria [13]. Although the difference is not statistically significant, the trend suggests improved outcomes with CP. A meta-analysis by Shwan et al. found similar results, with higher closure rates for tympanoplasty with CP compared to tympanoplasty with fascia. However, hearing outcome did not differ significantly in their cohort [21]. Other studies have reported similar findings, showing higher success rates in the CP group than in the fascia group [22,23]. This difference may be explained by the tendency of fascia to shrink and dehydrate postoperatively, which can lead to more residual perforations compared to type 1 tympanoplasty with CP [2]. Although some studies suggest that CP may reduce hearing improvement due to stiffness [24], cadaveric vibrometry techniques have shown only minor changes in hearing loss (8 dBHL or less) for frequencies below 8 kHz [25]. Likewise, when the cohort of children in this study operated with CP graft was compared to our previous cohort of children with fascia graft, no statistical difference was found in hearing outcomes. In this study, complications following tympanoplasty were observed in 45% of the total cohort, 38.9% of children, and 60% of adults. The most common complications observed were ear discharge and granulation tissue, which were also seen in the fascia cohort, where complication rates were comparable. However, all complications were minor and effectively managed with local antibiotics and corticosteroid drops. Based on our previous research [13], where a significant number of patients experienced purulent otorrhea, we have made it a standard practice to inform our patients postoperatively that in the event of purulent discharge, they should promptly consult our department. As a result, the number of cases of purulent otorrhea in our current cohort is notably high. At the final follow-up visit, complications persisted in only four patients (5%), including OME, granulation tissue, keloid formation, and numbness around the ear.

There have been discussions regarding the optimal age to perform tympanoplasty in children. In our study, there was no significant difference between adults (93.3%) and children (86.2%) in the success rate of tympanoplasty with CP (*p* = 0.45). We also found no difference between children 5–8 years and those older than 8 years, which aligns with findings from other studies [26]. Some studies suggest delaying surgery until a certain age and first performing an adenoidectomy to allow for Eustachian tube maturation [27]. However, in our study, age did not influence the success rate, and we performed tympanoplasty starting at the age of 5 years old. This surgery can also be performed at a younger age, but cooperation during postoperative care is limited and could be much more challenging. Therefore, we believe it is advisable to postpone the procedure until children have reached an age where they can cooperate sufficiently, unless hearing loss is pronounced with negative effect on speech and language development. Furthermore, the risk of acute otitis media and reperforation, is lower in older children compared to younger ones (<5 years) [28].

Perforations larger than 50% were associated with significantly lower closure and success rates in both the overall cohort and in children. Additionally, when the perforation was in the anterosuperior quadrant, the success rate was lower (80% vs. 96%), although this difference was not statistically significant. These findings suggest that larger and anterosuperior perforations may require careful graft selection, technique adjustments, and closer postoperative monitoring to improve outcomes. Patients should also be counseled on the higher risk of incomplete closure.

In this study, the proportion of children with a successful tympanoplasty 12 months postoperatively was significantly higher in those who underwent prior adenoidectomy compared to those who did not. These results suggest that adenoidectomy may increase the likelihood of achieving closed TM after tympanoplasty. It is known that removing adenoid tissue in children might alleviate obstruction of the Eustachian tube and improve middle ear ventilation. Salvis et al. found that adenoid hypertrophy was associated with lower success rates after tympanoplasty with fascia grafts [29]. Similarly, Ribeiro et al. identified previous adenoidectomy as a predictor of functional success in children older than 10 years old [30]. Other patient factors, such as allergies, a history of ventilation tubes, or risk factors such as cleft palate and ear discharge during surgery, did not predict successful outcomes.

Based on the findings of this study, we recommend performing tympanoplasty with CP over fascia considering the previously demonstrated benefits of CP. More specifically in children, we recommend the combination with adenoidectomy if adenoid hypertrophy is present to avoid observational delay. Furthermore, our study shows that tympanoplasty with CP in children up to 5 years old can be performed safely.

## 5. Limitations

This study had a relatively small sample size due to the exclusion of cases with otological comorbidities. While this decision helped maintain a more homogeneous study population, it may have influenced the outcomes. Additionally, no objective measurement was available for perforations, which may have introduced variability in the assessment and interpretation of the findings. Finally, it would have been interesting to analyze the primary pathology causing tympanic membrane perforation (e.g., otitis media, trauma, ventilation dysfunction), as different etiologies may influence surgical success rates.

## 6. Conclusions

In this study, type 1 tympanoplasty with CP in children demonstrated a high rate of TM closure (93.2%). Furthermore, successful surgery, defined as a closed TM and ABG < 20 dBHL, was achieved in 86.2% of the same group. Type 1 tympanoplasty outperformed the results from our prior study on tympanoplasty with fascia in children. No significant age-related differences in success rates were found, supporting the safety of tympanoplasty in children as young as five years old. Based on these findings, we recommend tympanoplasty with CP over fascia grafts in patients with TM perforations. Additionally, prior adenoidectomy was associated with higher success rates in children.

## Figures and Tables

**Table 1:** Influence of age on success rate tympanoplasty.

	Adults	Children	*p* Value
Closed TM ^1^ at 12 months	14/15 (93.3%)	55/59 (93.2%)	1
ABG ^2^ < 20 dB ^3^ postoperative	14/15 (93.3%)	54/58 ^a^ (93.1%)	0.97
Succes	14/15 (93.3%)	50/58 ^a^ (86.2%)	0.45
Mean change ABG (dB)	−6.1	−9.5	0.13
Mean preoperative ABG (dB)	18.3	18.4	1

^1^ TM: tympanic membrane. ^2^ ABG: Air–bone gap. ^3^ dB: decibel. ^a^: in one child no information on ABG postoperative was available.

**Table 2:** Outcomes of tympanoplasty by pediatric subgroups.

	<8 Years (%)		8–12 Years (%)		13–17 Years (%)		*p* Value
	*n*	%	*n*	%	*n*	%	
Closed TM ^1^ at 12 months	28/31	90.3	20/21	95.2	7/7	100	0.59
Succes	25/30	83.3	19/21	90.5	6/7	85.7	0.78

^1^ TM: tympanic membrane.

**Table 3:** Relation of patient factors and closed tympanum after surgery.

Patient Factor	Category	Success Rate (*n*, %)	*p* Value
Allergy	Yes	17/20 (85%)	0.12
	No	52/54 (96.3%)	
Revision	Yes	12/12 (100%)	0.58
	No	57/62 (91.9%)	
Contralateral ear	Normal	30/32 (93.7%)	1
	Abnormal	39/42 (92.8%)	
History of ventilation tubes	Yes	46/49 (93.8%)	1
	No	23/25 (92%)	
Risk factor	Yes	9/9 (100%)	1
	No	60/65 (92.3%)	
Ear discharge during surgery	Yes	11/12 (91.7%)	1
	No	58/62 (93.5%)	

This table presents the association between various prognostic factors and surgical success in the overall study population (children and adults).

**Table 4:** Relation perforation size and success rate tympanoplasty.

Perforation Size	Success Adults (*n*, %)	*p* Value	Success Children (*n*, %)	*p* Value
<25%	7/7 (100%)		20/20 (100%)	
25–50%	6/6 (100%)		31/32 (96.8%)	
>50%	1/2 (50%)	0.23	4/7 (57.1%)	0.002

**Table 5:** Complications after tympanoplasty.

Complications	Frequency (*n*, %)		
	Children	Adult	Total
Single ear discharge	11 (18.6%)	4 (26.6%)	15 (20.3%)
Granulation tissue	7 (11.8%)	1 (6.6%)	8 (10.8%)
Postoperative infections	5 (8.4%)	1 (6.6%)	6 (8.1%)
OME	4 (6.8%)	1 (6.6%)	5 (6.8%)
Keratinic pearl	0 (0%)	4 (20%)	4 (5.4%)
Numbness around ear	1 (1.7%)	1 (6.6%)	2 (2.7%)
Keloid formation	1 (1.7%)	0 (0%)	1 (1.3%)

OME: otitis media with effusion.

## Data Availability

The data presented in this study are available on request from the corresponding author due to privacy and ethical restrictions.

## Abbreviations

The following abbreviations are used in this manuscript:

ABG	Air–bone gap
AOM	Acute otitis media
dBHL	Decibel hearing level
CP	Cartilage-perichondrium
OME	Otitis media with effusion
PTA	Pure tone audiometry
TM	Tympanic membrane
